# Development of a biopsychosocial hybrid intervention to support patients following a complex lower-limb trauma: the PROMOTE study

**DOI:** 10.1302/2633-1462.711.BJO-2026-0153.R1

**Published:** 2026-11-03

**Authors:** Beth Fordham, Louise M. Quarmby, Emma Evans, Jonathon Merrill, Duncan Appelbe, Jenny Gould, Dominic Burton, Julie Wright, Jane Quinlan, Juul Achten, Elizabeth Tutton, Matthew L. Costa, David J. Keene

**Affiliations:** 1 Kadoorie Institute of Trauma, Emergency & Critical Care, Nuffield Department of Orthopaedics Rheumatology and Musculoskeletal Sciences, University of Oxford, Oxford, UK; 2 Oxford Major Trauma Centre, Oxford University John Radcliffe Hospital, Oxford University Hospitals NHS Foundation Trust, Oxford, UK; 3 Oxford Major Trauma Centre, John Radcliffe Hospital, Oxford University Hospitals NHS Foundation Trust, Oxford, UK; 4 NDORMS Patient and Public Involvement and Engagement group, Oxford, UK; 5 Trauma and Major Trauma Centre, Oxford University Hospitals NHS Foundation Trust, John Radcliffe Hospital, Oxford, UK; 6 Oxford University Hospitals NHS Foundation Trust, Oxford, UK; 7 University of Oxford, Oxford, UK

**Keywords:** Orthopaedic, Trauma, Biopsychosocial, Intervention development, Cognitive behavioural therapy, lower-limb trauma, Orthopaedic Trauma, clinicians, healthcare professionals, morbidity, randomized controlled trial, psychologists, physiotherapists, anxiety, complex fractures

## Abstract

**Aims:**

Complex lower-limb trauma is associated with substantial long-term physical and psychological morbidity. Access to integrated psychological support remains inconsistent. This study describes the development of Pain Rehabilitation to Optimize Major Orthopaedic Trauma REcovery (PROMOTE), a theory-based, hybrid (website plus one-to-one support) biopsychosocial intervention.

**Methods:**

Development followed the Medical Research Council framework for complex interventions and is reported in accordance with the GUIDance for the rEporting of intervention Development (GUIDED) checklist. This included: 1) an evidence review to identify modifiable psychosocial risk factors and theoretical models; 2) content coproduction with an expert advisory group including multidisciplinary clinicians and patient representatives, alongside independent patient interviews; and 3) iterative development and user testing of a low-intensity, scalable intervention. The intervention integrates the Common-Sense Model of Self-Regulation with cognitive behavioural therapy.

**Results:**

PROMOTE comprises four brief healthcare professional contact sessions delivered over eight weeks and an interactive, personalized website. The website is structured into five sections: 1) psychoeducation about the injury, pain, stress, and recovery; 2) cognitive behavioural techniques; 3) coping strategies; 4) recovery planning; and 5) a personalized profile. Content includes multimedia resources, interactive exercises, and personalized feedback. Iterative feedback from patients and clinicians led to improvements in navigation, language, pacing, and engagement.

**Conclusion:**

PROMOTE is a coproduced, theory-informed, scalable biopsychosocial intervention designed to address unmet psychological needs following complex lower-limb trauma. The intervention was evaluated in a feasibility randomized controlled trial to assess acceptability and engagement.

Cite this article: *Bone Jt Open* 2026;7(11):1398–1409.

## Introduction

Lower-limb fractures represent a major global health burden and are associated with substantial long-term disability, chronic pain, and psychological morbidity.^1,2^ Up to 63% of patients experience persistent pain,^3,4^ while depression, anxiety, and post-traumatic stress disorder are highly prevalent^5,6^ and may persist for several years.^7^ These factors are associated with delayed return-to-work,^7^ opioid misuse,^8,9^ reduced adherence to rehabilitation,^10^ and decreased health-related quality of life.^11^

Despite recommendations that major trauma centres (MTCs) provide integrated psychological care,^12^ provision remains inconsistent across the UK.^13,14^ Existing services often focus on screening and risk stratification rather than delivering scalable interventions. Consequently, there is a clear unmet need for accessible biopsychosocial support during recovery. This need is recognized in the James Lind Alliance Priority Setting Partnership,^15^ who included the following in their top ten research priorities for complex fractures: What psychological support would be useful for patients with complex fractures and when? What are the options for preventing and treating chronic pain after complex fractures? And What additional care and support is helpful for patients being discharged from hospital after a complex fracture?

The Pain Rehabilitation to Optimize Major Orthopaedic Trauma REcovery (PROMOTE) intervention was developed to address this gap by providing a scalable, hybrid (face-to-face and digital) self-management intervention. This paper describes its systematic development.

## Methods

PROMOTE was developed from May 2022 and September 2023 as a collaboration between clinicians, researchers, and patient representatives. Development followed the Medical Research Council framework,^16^ and is reported in line with GUIDance for the rEporting of intervention Development (GUIDED) recommendations.^17^

### Evidence review

Psychosocial factors are stronger predictors of poor recovery than injury severity following lower-limb trauma.^18^ The risk factors include: 1) sociodemographic variables: older age, being female, being from an ethnic-minority group (based on studies in Europe and USA where White is the ethnic-majority group), lower economic status, fewer years of education, and having low levels of social support;^19-21^ 2) cognitive variables: low pain self-efficacy, high pain catastrophizing, high fear-avoidance, and low resilience;^22-24^ 3) emotional variables: pre-injury anxiety and depression and post-injury anxiety, depression, and post-traumatic stress disorder;^10^ and 4) early injury symptoms: e.g. severe initial pain, sleep, and rest dysfunction.^19,20^

Cognitive variables can be modified using psychological techniques, which have been successfully used to produce clinically significant improvement in pain and disability outcomes in chronic pain populations.^25,26^ Therefore, we aimed to use psychological models to develop an intervention which can promote adaptive cognitions (i.e. high pain self-efficacy) as alternatives to unhelpful cognitions (i.e. high fear-avoidance). By adopting a biopsychosocial design, the PROMOTE intervention also included elements to optimize early pain management and sleep/rest dysfunction in addition to targeting cognitive variableCommon-Senses.

### Theoretical model

The Common-Sense Model of Self-Regulation (CSM-SR)^27^ describes how perceptual, cognitive, and behavioural processes interact in the self-management of illness or injury. The model includes seven cognitive representations — identity, timeline, consequences, causes, personal control, treatment control, and coherence — which influence emotional responses (e.g. anxiety or stress) and coping behaviours. Figure 1 presents example beliefs associated with complex lower-limb orthopaedic trauma. For instance, if a patient believes physiotherapy will not control their pain (low treatment control), they may be less likely to adhere to rehabilitation exercises. Similarly, pain-related fear during movement may lead to fear-avoidance behaviours, such as stopping activity when pain occurs.

**Fig. 1:**
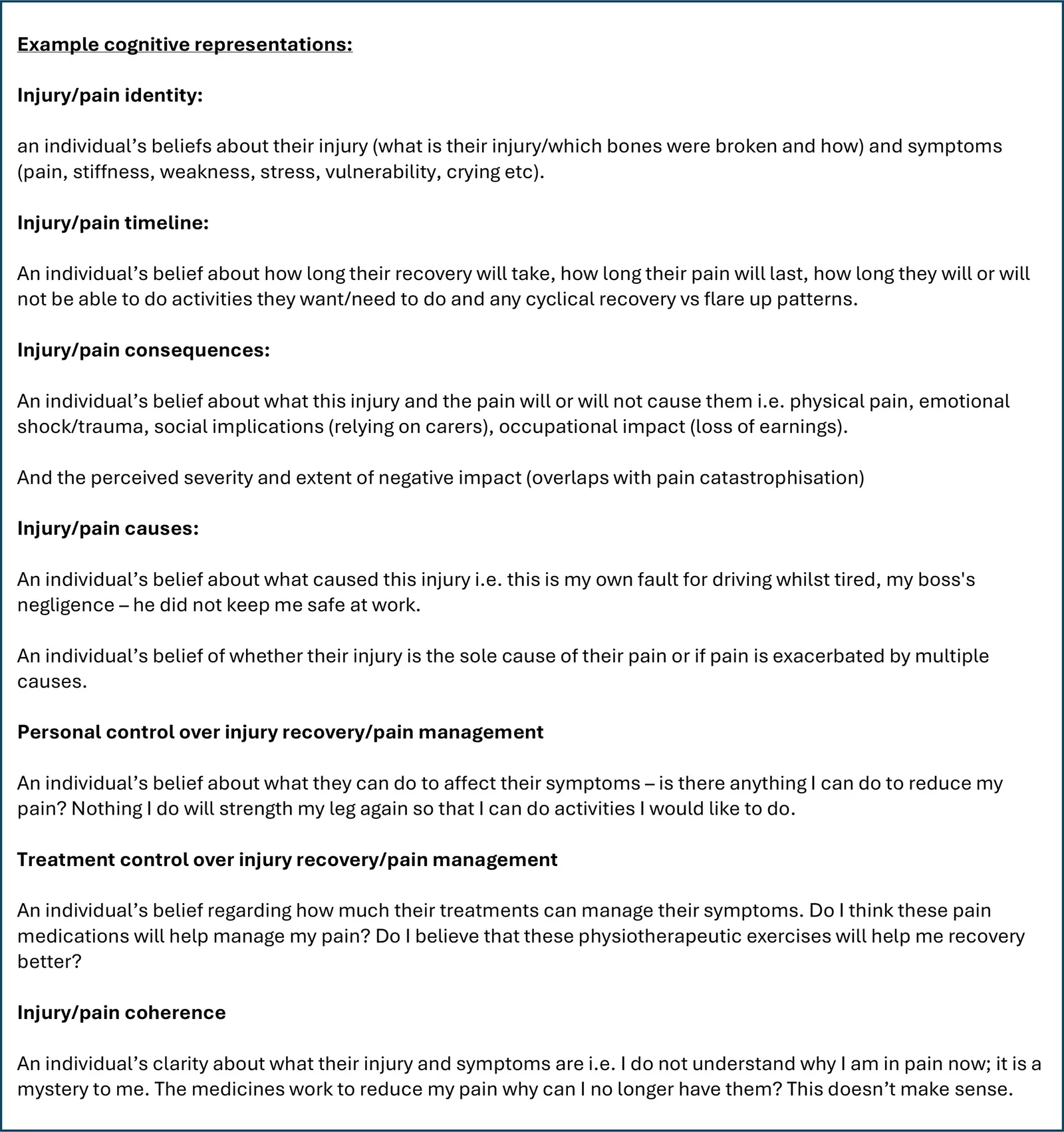
Example cognitive representation of a complex lower-limb orthopaedic trauma.

### Therapeutic model

The CSM-SR describes injury representations but does not specify a therapeutic intervention. Therefore, it was necessary to translate the model into an intervention that could be delivered at scale across UK MTCs.

Clinical psychologists Dr Quarmby and Dr Evans developed the outline cognitive behavioural therapy (CBT)-based psychoeducational pain management tool at the Oxford MTC. This was presented to the National Major Trauma Psychology Network, whose members agreed that CBT was an acceptable and appropriate intervention within UK MTCs.

CBT has a strong evidence base for improving outcomes such as quality of life, anxiety, depression, PTSD, and pain.^26^ Consequently, CBT was selected as the underpinning therapeutic model for the PROMOTE intervention. Given the limited availability of full-time clinical psychology support within UK MTCs, the intervention was designed primarily as a self-management resource.

The CSM-SR has previously been successfully translated onto the CBT delivery model in other health conditions.^28^Figure 2 represents how CBT techniques can be mapped onto the CSM-SR.

**Fig. 2:**
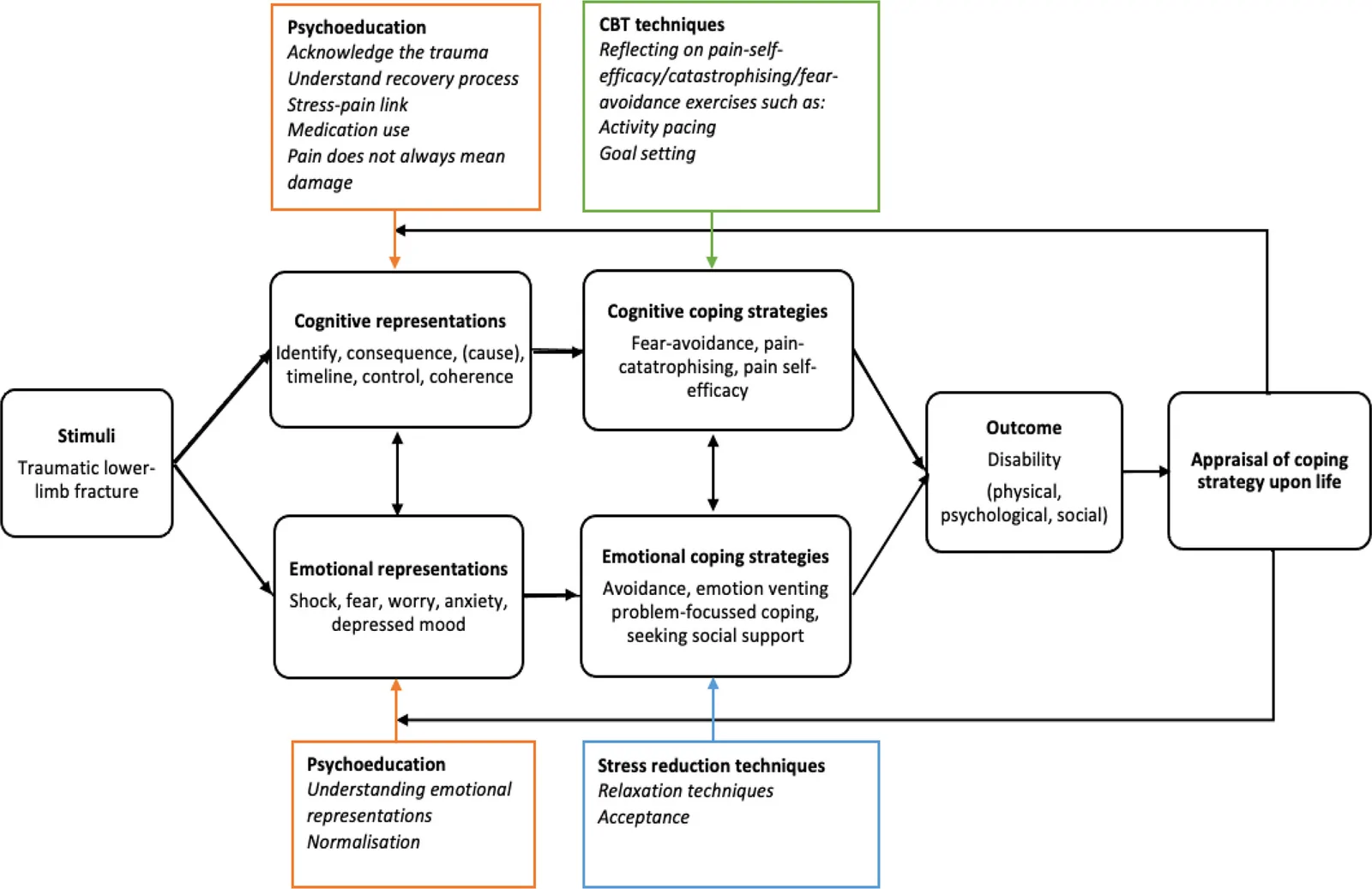
The Pain Rehabilitation to Optimize Major Orthopaedic Trauma REcovery (PROMOTE) theoretical model - mapping cognitive behavioural therapy (CBT) onto the Common-Sense Model of Self-regulation (CSM-SR) model for complex lower-limb orthopaedic trauma.

### Codevelopment

The expert advisory group (EAG) codeveloped the intervention content, and stakeholders included:

- BF led on the development of the psychological components of the intervention, including content and writing patient-facing materials. Two clinical psychologists (LMQ, EE) from the Oxford Psychological Medicine Service contributed a clinical perspective. These members were experts in the psychological tools they use in their clinical work with patients with major trauma and pain.
- Two consultant orthopaedic trauma surgeons (MLC, SG) from the Oxford MTC. These members guided the medical perspectives of the trauma and recovery.
- One consultant nurse from the Oxford MTC (JW). To advise on the daily experience of patients and the care and advice work which helps patients.
- An Advanced Practice Physiotherapist (DJK) to advise on physical rehabilitation.
- Two patients (JG, DB) who have experienced this injury and offered the patient perspective on what was helpful or unhelpful during their recovery.
- A consultant anaesthetist (JQ) from the Oxford University Hospitals NHS Foundation Trust for guidance on how patients can optimize their pain medication management.

The author (BF) met and interviewed members of the EAG to discuss and refine the content of the PROMOTE intervention over a period of 12 months.

Patient and public patient involvement and engagement (PPIE) representatives recommended additional interviews with patients not involved in the EAG to capture perspectives independent of the PROMOTE research aims. Three patient representatives were interviewed in June 2022. The following categories were identified: injury and recovery journey, medication, pain, social support, emotions, transition to outpatient care, occupational challenges, pain management, and sleep (Table I).

**Table I.** Field notes of patient interviews regarding the intervention content.

Content	Field notes
Injury journey	“Chaos” confusing in trauma ward Clear differentiation between “injury” and “recovery” stage Terrifying and thought “I am going to die”
Medication	Catastrophizing “not in control” and “this will always be this painful” “No idea what I was taking” Did not like taking so much pain medication as was in dissonance with their self-image of a ‘healthy, strong person’
Pain	Important to understand pain as early as possible Wish he’d have known then what he knows now The pain was manageable but the mental disturbance was the main problem
Social support	Social support is critical Raised the question of if the advice from a patient’s family/friends is in opposition to medical advice (religion/culture) Carers need to be acknowledged
Inpatient emotions	Anxious, frustrated, frightened, overloaded with information “No one talked to me” Dark, “Self-pitying”, “When will I be back and normal again?” Embarrassed to be struggling “put on a brave face” Uncontrollable crying, depressed, angry. Bitter, “I set unrealistic goals that I would be back walking in 8 weeks. When this passed, I felt even worse”
Outpatients’ emotions	Sinking in, anxiety “life will never be the same” Anger, acceptance of the new normal, found motivation Needed more psychological support when he got home
Occupational/legal	Huge burden for one patient
Pain management	Uses pain as an excuse when mood is low Distraction to manage pain flares Male pride stopped me asking for help Changed self-perception from strong independent to a person who needs to ask for help
Sleep	Not a problem
Information retention	Forgot what they had been told, need a place to write down questions when they think of them in order to ask the healthcare professional at their next meeting.
Key messages for PROMOTE development	Important to normalize the feelings of anger and worry Maybe one website for in-hospital and one for at-home? Must be user-friendly and engaging Early stages must be simple and little information Likes the idea of leaving a message to the HCP More knowledge makes a patient feel empowered to ask more questions Make sure there is information for carers/family to help start the conversations Acknowledge the carer’s shock Goals must be meaningful to my life Expectation management Writing things down is very helpful “Take time to allow yourself to refuel” message

HCP, healthcare professional; PROMOTE, Pain Rehabilitation to Optimize Major Orthopaedic Trauma REcovery.

The EAG reviewed field notes from the patient interviews alongside the evidence review. Members of the EAG led development within their areas of expertise. For example, clinical psychologists developed some resources to support patients with pain and injury-related cognitions; the anaesthetist produced a video explaining pain medication use; the surgeon explained bone healing and how movement and strengthening support recovery; the nurse addressed the inpatient experience and transition to discharge; patients recorded a talking-head to explain how and why this tool can help in recovery, and the physiotherapist focused on physical rehabilitation such as graded activity. The process was overseen by BF to ensure continuity across all materials.

The EAG recommended: 1) using a range of communication strategies (infographics, animations, videos, simple text, and question-and-answer formats); 2) including an interactive element on the website where participants could record questions or reflections between appointments; 3) providing one-to-one support to help participants engage with the website, particularly as it represented a new approach within recovery care; 4) bridging the inpatient to outpatient transitions as this can be a particularly challenging time for patients; and 5) developing a training package, including session crib sheets, for staff delivering the one-to-one support sessions.

### Iterative development and testing

The intervention was translated into a digital platform and iteratively refined. The website draft was fully reviewed by our clinical psychology EAG members; their feedback and our amendments are presented in Table II.

**Table II.** Clinical psychology expert advisory group member feedback to draft Pain Rehabilitation to Optimize Major Orthopaedic Trauma REcovery (PROMOTE) website.

Feedback	Field notes	Amendments
Pain psychoeducation	The pain psychoeducation section was noted to be too onerous for patients, especially in the early days post-trauma.	Animation software (VideoScribe) was employed to develop visual animations to communicate the pain system conceptions.
Medication education	It is essential that we are giving clear information about medication suitability changing over time from the outset.	Videos of an anaesthetist explaining the key concepts of pain medication were included.
Stress reduction section	Mindfulness is not relaxation and should not be introduced as such. Stress reduction is a more appropriate term.	Amended throughout
Activities to support recovery section	Empowerment will come from patients learning how to use positive/helpful coping strategies regardless of internal experiences – it is the actions/behaviours that should be focused on here.	Ensured the activities to support recovery section is focused on helpful coping actions/behaviours.
Cognitive behavioural therapy section	The language here does not feel patient-friendly, empathic, or engaging.	Changed the language to more conversational and included patients quotes.
Pain management strategies	Encouraging the monitoring of pain is not something we would encourage.	Removed the physiotherapy behaviour change section in which pain monitoring was suggested.

We also sought staff and patients external to the EAG for objective feedback. The draft was reviewed by pain management staff and clinicians from two other MTCs (University Hospital Cambridge and University Hospitals Coventry and Warwickshire NHS Trust). And, the same three patients who had been interviewed during the content development phase (Table III).

**Table III.** Patient feedback to draft Pain Rehabilitation to Optimize Major Orthopaedic Trauma REcovery (PROMOTE) website.

Feedback	Field notes	Amendments
Primary concern	The website navigation is not clear/needs explicit instruction The user must understand that they do not work through all content at once Concern that the content was too much at the early stage of recovery	Focus on improving navigation Explainer videos embedded into the website Included step-by-step messaging into face-to-face sessions
Wording/style	Prefers conversational style Concerned that the initial introduction pages were too slow Still a bit basic would prefer an app Clear and easy to understand	Reduced the initial introduction pages. Include conversational patient quotes.
Clarification section	Less relatable because it was more medical Suggested quote “Ask a question, it is never silly” Loved the reassurance to ask lots of questions	Included patient quotes throughout such as “Ask a question, it is never silly”
Cognitive behavioural therapy section	This section is tricky but once understood it is worthwhile and relatable Acknowledges that their mind made their pain worse Getting to know your pain and accepting it	
Activities to support recovery section	Reiterate that this is not something you have to do, this website and the exercises are for you to help your recovery The breathing exercises felt very relevant to managing the pain	Include reassurance that the website is something the patients can do but they do not have to do in the face-to-face sessions.
Physiotherapy behaviour change section	Really struck a chord They really got to understand “where the physios were coming from” Loved making goals Liked all the simple patient quotes – helped make it all relatable The videos helped engagement	Include conversational patient quotes. Include more videos to communicate the messages

The final version of the website underwent usability testing prior to launch. At this stage, formal testing was undertaken and all the documentation required to comply with best practice within the software engineering industry and the University of Oxford Clinical Trials Research Unit (OCTRU) standard operating procedures (SOPs).

## Results

PROMOTE is a hybrid intervention combining HCP-supported sessions with a self-directed digital platform.

### Healthcare professional sessions

Participants are offered four sessions with a PROMOTE HCPS over eight weeks either inpatient or remote access outpatient. Sessions aim to introduce the intervention, support engagement, address barriers, and reinforce self-management strategies.

### Website structure and content

The website comprises five sections:

1. What is happening to me?: This section aimed to: 1) acknowledge and normalize participants’ emotional responses to complex orthopaedic trauma; 2) enhance personal and treatment control by supporting transparent communication between participants and HCPs about the injury (addressing the identity of the CSM-SR), treatments, and medication; and 3) improve injury coherence through psychoeducation on the biopsychosocial nature of pain and pain medication use. A screnshot for this section of the website is shown in Figure 3.

**Fig. 3:**
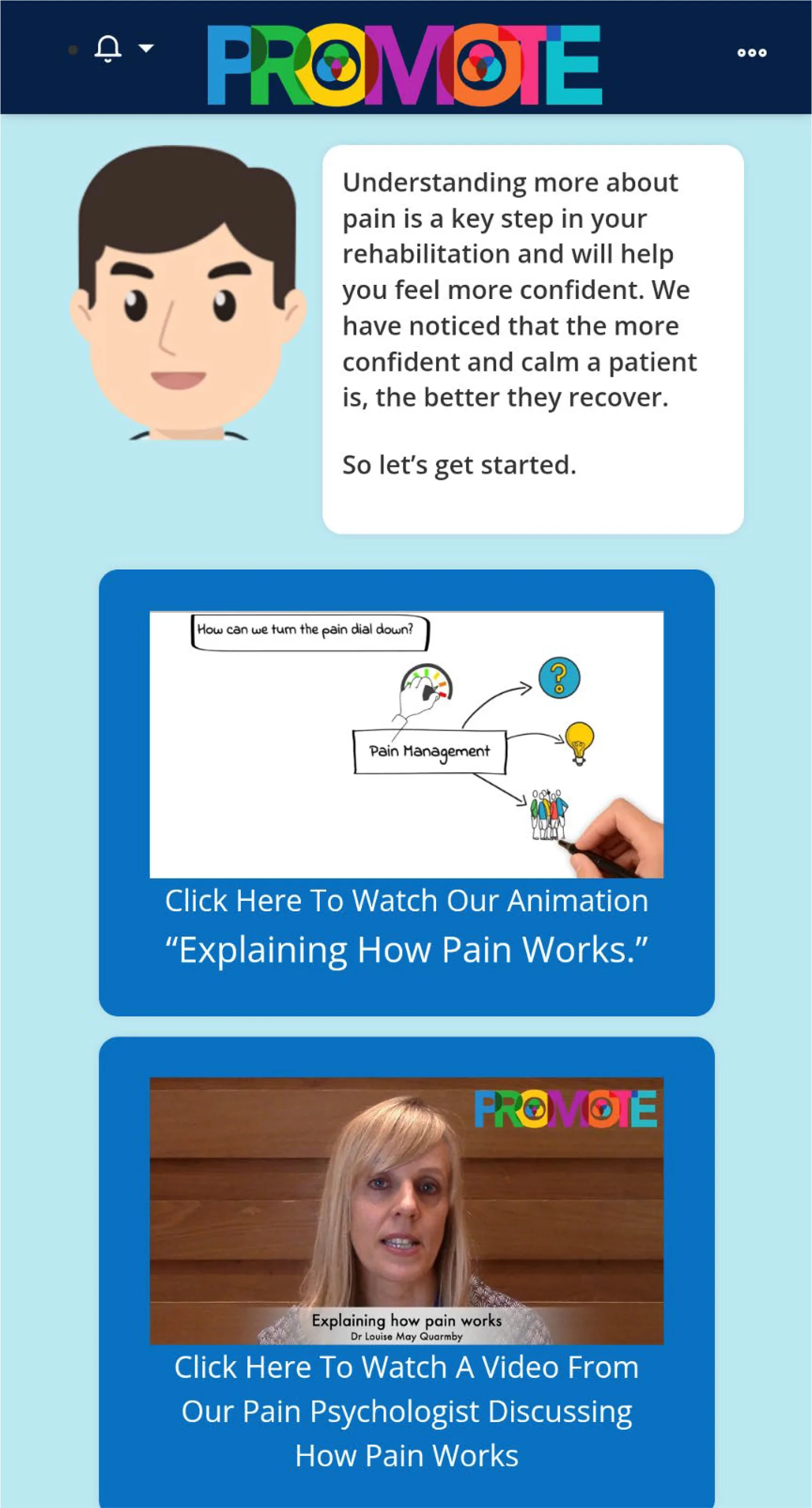
Screenshot from "What is happening to me section?".

HCPs entered details of the participant’s injury, treatment, and medication into a personal profile using clear, jargon-free language. Participants could review this information and submit questions via the website using text or audio messages. HCPs responded either through the platform or during subsequent one-to-one sessions. Encouraging questions aimed to clarify injury beliefs while fostering agency and personal control in recovery self-management.

A dedicated section addressed pain medication and targeted treatment control beliefs. This included videos from a consultant anaesthetist explaining common concerns about pain treatment, such as the rationale for taking medication before rehabilitation exercises to help stabilize pain levels. The materials also explained, in accessible language, why certain medications (e.g. opioids) may be used during acute pain phases but are not recommended for long-term use.

Another core component focused on understanding pain, addressing pain identity, causes, consequences, and coherence. The content introduced pain as a multifaceted biopsychosocial experience rather than solely a signal of tissue damage. Multiple communication formats — animations, video question-and-answer segments, and patient quotations — were used to convey these concepts, reflecting priorities identified by the EAG.

Recovery expectations and timeline representations were briefly introduced here and explored in greater depth in the fourth stage of the website.

2. How will my mind and body recover?: This section aimed to normalize participants’ experiences and explain how both physical and psychosocial factors influence injury and recovery. It focused on stress and how the participant’s situation may involve multiple stressors, such as occupational, social and family pressures, or reduced access to stress-relieving activities (injury consequences), as well as the interaction between pain and stress (injury coherence).

The website introduced how participants might think (cognitive), act (behavioural), feel emotionally, and respond physically when experiencing pain and stress during recovery. Examples were drawn from patient-representative quotations alongside ‘imaginary patients’, based on staff and patient experiences.

Within this section, several CBT coping strategies were introduced, focusing on three psychological risk factors associated with chronic pain in this population: pain self-efficacy, pain catastrophizing, and fear-avoidance. Common unhelpful thoughts were presented alongside video responses from clinical experts offering alternative, more adaptive interpretations. For example, an imaginary patient might state, “I might make my pain worse if I move” (fear-avoidance). In response, a consultant surgeon explained that movement is safe and an expected part of rehabilitation, helping to normalize concerns and support pain self-efficacy (Figure 4).

**Fig. 4:**
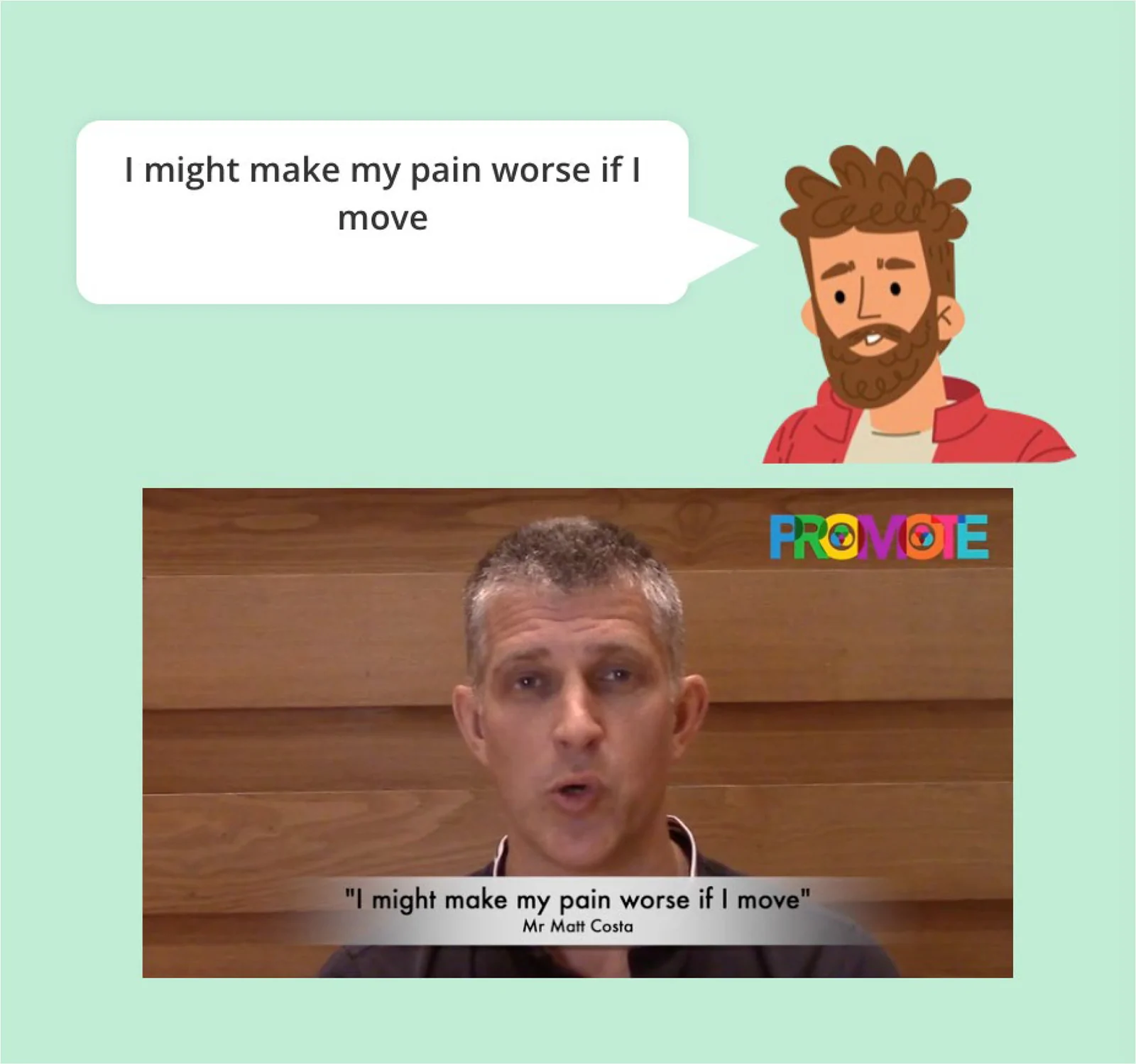
Example of unhelpful thought and reassurance from a healthcare professional.

These examples encouraged self-reflection and presented alternative beliefs that patients could adopt, particularly those that sit between extreme or dichotomous interpretations often associated with pain catastrophizing.

Participants were also introduced to common CBT ‘thought patterns’, such as mind-reading. The website provided examples reported by patients or observed by clinical psychologists (e.g. “My nurse will think I am annoying if I ask the same question again”). Identifying and labelling these patterns aimed to help participants recognize that such thoughts are not necessarily factual, thereby supporting greater personal control and more flexible ways of thinking.

3. What can I do to feel better?: Section three offered adaptive cognitive and emotional coping strategies such as:

- Stress reduction audio-recordings exercises (ten to 30 minutes), which include visualizations, present moment awareness, and breath work (Figure 5). Participants could save any exercises they liked into their personal profile.
- Activities to improve sleep while in hospital and when participants have returned home.
- Tips to help participants structure their days to avoid stress and other emotional responses becoming overwhelming.

**Fig. 5:**
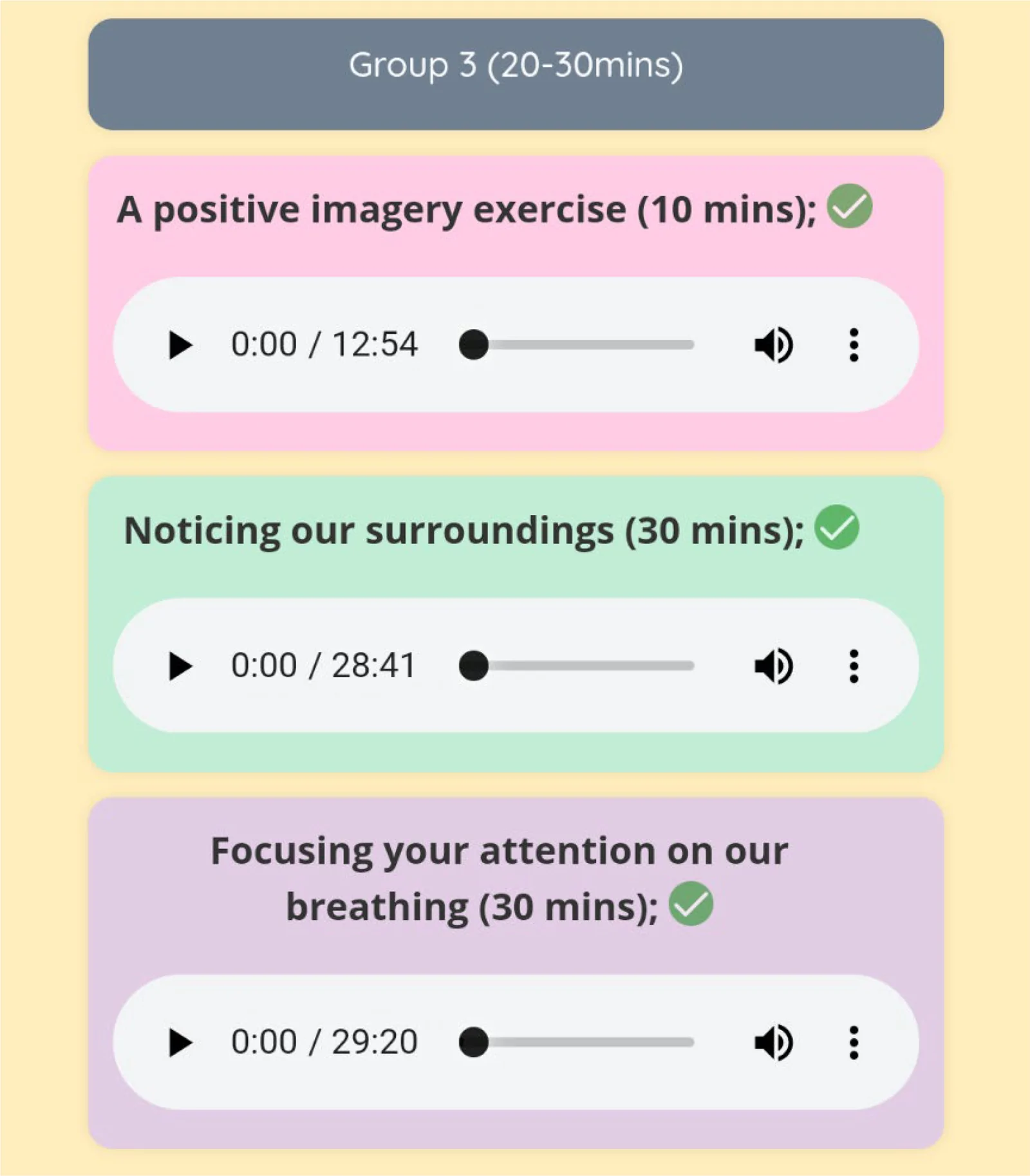
Example of bespoke stress reduction audio exercises.

4. Where to next?: This section was predominantly informed from physiotherapists and implements problem-focused coping strategies to manage high fear-avoidance, high pain catastrophization, and low pain self-efficacy beliefs. These coping strategies included value-based specific, measurable, attainable, realistic, time-bound (SMART) goal setting,^29^ setting activity baselines, graded increases, and pacing to avoid ‘boom and bust’.

This section of the website aimed to support adaptive behaviour change by increasing participants’ beliefs in their personal control, the physiotherapy treatment control, and managing recovery timeline expectations. We used a mixture of videos from experts and patients, infographics, and information registries to communicate the adaptive coping strategies.

5. PROMOTE profile: The PROMOTE profile was a self-management tool. All the answers which participants selected, the exercises they saved, and their personal notes were assimilated into their profile. When the participants return to the profile, they were able to focus on content which was specifically pertinent to them.

The profile had a function which allowed participants to choose if they would like reminders sent (SMS/email) at certain times of the day. The reminder would identify the coping strategy the participant chose (e.g. a sleep strategy), why they chose it, and what barriers and facilitators might have influenced if they used it or not.

## Discussion

The need for psychological support in orthopaedic trauma recovery has been highlighted by the James Lind Alliance priority setting exercise^15^ and the Major Trauma Psychology Network. The PROMOTE intervention was developed using theory and evidence, alongside input from multidisciplinary HCPs and patients who coproduced and iteratively refined the content until consensus was reached.

The intervention was implemented as an interactive website, supported by a training programme enabling HCPs to deliver four one-to-one sessions, aimed at encouraging engagement with the platform. While website usability was evaluated, the effectiveness of the staff training was not formally assessed.

Audio and video materials were recorded using professional equipment to ensure high-quality content. However, the website itself was developed without a professional production company, as this feasibility study aimed to evaluate the intervention content prior to investing in a more costly mobile application.

A limitation of this development process was that while we included perspectives from multiple healthcare professionals, these were from only five hospitals. We successfully expanded this group in our feasibility study by working with three additional hospitals from different regions of England, to assess feasibility in a range of major trauma centres.

Prior research studies with trauma populations have found it hard to retain participants in clinical trials, and as this is a novel intervention for the patient population, we may encounter problems with retention, hence the need for a robust feasibility study.

Findings from the feasibility evaluation^30^ indicated that although PROMOTE content was valued by patients, a more accessible and engaging mobile application will likely be required to support sustained patient engagement.

**Take home message**

- Pain Rehabilitation to Optimize Major Orthopaedic Trauma REcovery (PROMOTE)'s clinical relevance lies in its biopsychosocial approach to recovery after major lower-limb trauma, addressing the complex physical, psychological, and social challenges that can influence rehabilitation and longer-term outcomes.

- By providing a comprehensive and transparent account of its methodological and theoretical foundations, this paper supports clinicians in understanding how PROMOTE was developed and its potential role in delivering structured, patient-centred support throughout recovery.

## Data Availability

The data supporting the findings of this study are available from the corresponding author upon reasonable request.
